# Research note: Peripheral administration of Met-enkephalin: Novel attenuation of the adrenal medullary and thyroidal stress responses in young female chickens

**DOI:** 10.1016/j.psj.2025.105748

**Published:** 2025-08-28

**Authors:** Colin G. Scanes, Krystyna Pierzchala-Koziec

**Affiliations:** a University of Wisconsin Milwaukee, USA; b University of Agriculture, Krakow, Poland

**Keywords:** Met-enkephalin, Catecholamines, Thyroxine, Triiodothyronine, Pullets

## Abstract

Adrenal hormones play a critical response to stresses in poultry, livestock and other vertebrates. Moreover, there are stress effects on thyroid hormones. Plasma concentrations of epinephrine (EP), norepinephrine (NE), thyroxine (T_4_) and triiodothyronine (T_3_) were increased by restraint stress (placing pullets into a small cage in darkness) in 14-week-old pullets. There has been a complete dearth of publications on the effects of peripheral administered Met-enkephalin on endocrine systems in chickens. To remedy this, the effects of peripheral administration of Met-enkephalin *per se* was examined as was along with its ability to either potentiate or attenuate the responses to stress. Plasma concentrations of EP and NE were modestly increased in chickens receiving Met-enkephalin injection peripherally. The increase in plasma concentrations of EP, NE, T_3_ and T_4_ together with the decrease in reverse T_3_ in chickens subjected to restraint stress were markedly attenuated in pullets receiving Met-enkephalin injection peripherally.

## Introduction

Stressed animals have elevated plasma concentrations of glucocorticoids and catecholoamines; these hormones being produced by, respectively, cortical and medullary cells in the adrenal glands (reviewed Carsia et al., 2022). Plasma concentrations of adrenal neuropeptide, Met-enkephalin, are also increased by stressors, for instance, in chickens (Scanes et al., 2024); Met-enkephalin [tyrosine–glycine-glycine- phenylalanine–methionine (YGGFM)] being produced in the adrenal chromaffin and probably cortical cells (Scanes et al., 2024). It was hypothesized that Met-enkephalin plays a modulating role in stress responses.

Walter Cannon developed the concept of “fight or flight” with animals responding to an acute threatening situation with release of epinephrine (**EP**) and norepinephrine (**NE**) (reviewed: Goldstein, 2010). "*For decades, catecholamines have been used as a measure of stress in animals*” (Dennis, 2016). While “fight or flight” is frequently mentioned in poultry literature (reviewed Carsia et al., 2022), there is a dearth of studies reporting effects of stresses on plasma concentrations of EP and NE in chickens. An exception to this is the report that plasma concentrations of EP but not those of NE were increased post-stunning in broiler chickens (Riggs, 2022). Parenthetically, Dennis (2016) concluded that over 90 % of EP originates in the chromaffin cells while less than 10 % of NE does. The present study examines the effects of restraint stress on plasma concentrations of glucose, EP and NE and the extent to which peripherally administered Met-enkephalin either augments or attenuates any stress related increases; restraint stress being a widely employed model for stress in chickens (e.g. Kuenzel et al., 2014; Kadhim et al., 2020). It was hypothesized that plasma concentrations of EP and NE would be increased in pullets subjected to restraint stress.

Plasma concentrations of triiodothyronine (**T_3_**) have been reported to be decreased while those of thyroxine (**T_4_**) were increased in chickens following either heat stress (Beckford et al., 2020) or withholding feed (Van der Geyten et al., 1999). Moreover, there are increases in the expression of thyroid hormone related-genes (e.g. thyrotropin β sub-unit and thyroid receptor β) in the pituitary gland of heat stressed chickens (Beckford et al., 2020). There is, however, little other evidence that stresses influence plasma concentrations of thyroid hormones. What is also not known is whether Met-enkephalin influences plasma concentrations of T_4_, T_3_, and reverse T_3_ (**rT_3_**) in stressed and non-stressed chickens. This is examined in the present study.

## Materials and methods

### Materials and methods

All animal procedures were conducted with prior institutional ethical approval in accordance with the Local Institutional Animal Care and Use Committee (IACUC). The animal study was approved by the Institutional Review Board and the First Local Ethical Committee on Animal Testing in Krakow, Poland (protocol 120/2013). The experiment was conducted in accordance with the principles and specific guidelines presented in Guide for the Care and Use of Agricultural Animals in Research and Teaching, 4th edition, 2020.

### Animals

The studies were performed on 14-week-old female chickens (line - ISA Brown), weighing 1.30 ± 0.10 kg. During the acclimatization period (7 days), the birds were kept in individual cages (60 × 60 × 40 cm) with commercial feed and water available *ad libitum*. The chickens were maintained in a controlled environment with a temperature of 20°C and a photoperiod of 12L:12D (lights on from 7.00 a.m. to 7.00 p.m.). Care taken to ensure that the birds were handled gently in view of the stress response to rough handling (Chloupek et al., 2011).

### Experimental design – Effect of opioid on the attenuation of adrenal medullary and thyroidal responses to restraint stress

There were four treatment groups (five chickens per group): 1. controls that were individually caged pullets and receiving vehicle administration, 2. Met-enkephalin (endogenous delta receptor opioid ligand) injected i.v. (1mg/kg b.w.), 3. stressed by 30 min of restraint (pullets moved to small cages 30×30×40 cm in darkness) and receiving vehicle administration, and 4. birds receiving Met-enkephalin and then subjected to restraint stress.

### Blood sampling

Blood samples (2 ml) were obtained by venipuncture from the right branchial vein at the following times: 5 min before treatment, immediately prior to injections and stress (marked as −1′), 15′, 30′ (at the end of the imposition of stress) and 60′ after the start of experiment. The blood samples were divided into two sub-samples in polypropylene tubes containing either heparin (1000 IU) for glucose, T_3_, T_4_, and rT_3_ or EDTA 2.7 nmole mL^−1^ together with citric acid (17.7 nmole mL^−1^) and aprotinin (Trasylol, 200 KIU mL^−1^) for EP and NE. After centrifugation (30 min at 4°C and 4000 × *g*), plasma was stored at −80°C until further processing.

### Hormone assays

Catecholamines were determined by enzyme immunoassay for the quantitative determination of EP and NE in plasma (3-CAT ELISA ^Fast Track^, Cat. BA E-6600, LDN, Germany). The standard range was: EP 0/1 – 200 ng/ml; NE: 0/5 – 1,000 ng/ml; sensitivity: EP 10 pg/ml plasma; NE - 36 pg/ml plasma. Plasma concentrations of thyroid hormones were determined by the following radioimmunoassays: 1. rT_3_ –Dia-Source (Belgium, cat. R-EW-125), standard range 0.02 – 2.14 ng/ml, sensitivity 0.014 ng/ml; 2. Total T_3_: DRG (Germany, cat. RIA-4534), standard range 0.35 - 14.0 nmol/l, sensitivity 0.22 nmol/l and 3. Total T_4_: DRG (Germany, cat. RIA-4533) standard range12.8 - 500 nmol/l, sensitivity 5.0 nmol/l.

### Glucose assay

Glucose concentration in plasma was estimated by kit Pointe Scientific, Glucose (Hexokinase) Liquid Reagents for *the in vitro* quantitative measurement of glucose in plasma (G7517120, Canton, MI, USA).

### Statistics

Data for time series in the same animals were analyzed by ANOVA for repeated measures. Data for the sampling 15-minute post-initiation of stressing/injection of Met-enkephalin or vehicle and for delta (increase or decrease) 15-minutes following treatment minus mean pre-treatment concentrations (−1 and −5′) were analyzed by two-way ANOVA. Means were separated by Tukey’s honest significance test as the range test.

## Results and discussion

### Met-enkephalin and/or restraint stress on plasma concentrations of EP and NE

Plasma concentrations of EP were increased in pullets receiving Met-enkephalin (by 42.0 %, *P* < 0.001) or subjected to restraint stress (by 92.2 %, *P* < 0.001) (Table 1). In contrast, there were decreased plasma concentrations of EP (by 37.5 %, *P* < 0.001) in pullets receiving both Met-enkephalin and restraint stress (Table 1). Similarly, plasma concentrations of NE were increased (*P* < 0.001) in pullets receiving Met-enkephalin (42.0 %) or subjected to restraint stress (by 78.8 %, (Table 1). Again, the effect of restraint stress was markedly attenuated when Met-enkephalin was injected (by 39.4 %, *P* < 0.001) (Table 1).

**Table 1:** Effect of peripheral administration of restraint stress and/or i.v. administration of Met-enkephalin (1 mg kg b.wt.^−1^) on plasma concentrations of epinephrine (EP), norepinephrine (NE), glucose, thyroxine (T_4_), triiodothyronine (T_3_) and reverse T_3_ (rT_3_).

	Time in minutes				
	−5	−1	+15	+30	+60
**Epinephrine (EP) nmoles L^−1^**					
Control (vehicle)	6.7 ± 0.09	7.1 ± 0.09	6.4 ± 0.15^a^	6.4 ± 0.16	6.0 ± 0.11
Met-enkephalin	6.2 ± 0.09^x^	6.7 ± 0.12^x^	9.1 ± 0.10^yc^	7.1 ± 0.09^x^	7.0 ± 0.13^x^
Stress (+ vehicle)	5.9 ± 0.11^x^	6.1 ± 0.07^x^	12.3 ± 0.23^zd^	7.1 ± 0.12^y^	6.1 ± 0.14^x^
Stress + Met-enkephalin	6.0 ± 0.10^x^	6.7 ± 0.13^y^	7.7 ± 0.14^zb^	5.0 ± 0.11^w^	3.8 ± 0.11^v^
**Norepinephrine (NE) nmoles L^−1^**					
Control (vehicle)	4.5 ± 0.08^x^	5.3 ± 0.12^y^	4.2 ± 0.15^xa^	3.9 ± 0.10^x^	4.2 ± 0.10^x^
Met-enkephalin	4.8 ± 0.11^x^	4.9 ± 0.09^x^	7.4 ± 0.17^yc^	4.8 ± 0.17^x^	4.7 ± 0.29^x^
Stress (+ vehicle)	4.2 ± 0.10^x^	4.0 ± 0.07^x^	10.2 ± 0.19^zd^	5.1 ± 0.24^y^	4.9 ± 0.14^y^
Stress + Met-enkephalin	4.4 ± 0.07^x^	4.4 ± 0.09^x^	6.2 ± 0.11^yb^	8.7 ± 0.16^z^	4.0 ± 0.14^x^
**Glucose mg dL^−1^**					
Control (vehicle)	186 ± 1.9^x^	188 ± 1.0^xy^	193 ± 2.0^za^	190 ± 1.7^y^	195 ± 1.7^z^
Met-enkephalin	192 ± 1.7^x^	198 ± 1.0^y^	199 ± 1.5^ya^	196 ± 2.2^y^	203 ± 2.2^z^
Stress (+ vehicle)	189 ± 1.4^x^	196 ± 2.0^y^	270 ± 1.5^zb^	272 ± 1.6^z^	203 ± 3.2^y^
Stress + Met-enkephalin	196 ± 1.5^x^	197 ± 1.5^x^	264 ± 1.7^zb^	257 ± 2.4^y^	200 ± 1.7^x^
**Thyroxine (T_4_) nmoles L^−1^**					
Control (vehicle)	16.0 ± 0.09^x^	16.8 ± 0.17^y^	16.7 ± 0.11^ya^	16.7 ± 0.12^y^	16.0 ± 0.12^x^
Met-enkephalin	15.8 ± 0.09^x^	16.5 ± 0.17^z^	16.1 ± 0.11^ya^	16.7 ± 0.12^z^	15.7 ± 0.13^x^
Stress (+ vehicle)	15.8 ± 0.13^y^	16.1 ± 0.13^y^	18.3 ± 0.16^zc^	15.4 ± 0.16^x^	14.8 ± 0.17^w^
Stress + Met-enkephalin	16.0 ± 0.09^x^	16.8 ± 0.07^y^	17.6 ± 0.23^zb^	17.3 ± 0.55^yz^	15.7 ± 0.14^x^
**Triiodothyronine (T_3_) nmoles L^−1^**					
Control (vehicle)	3.2 ± 0.19^x^	3.3 ± 0.11^x^	3.6 ± 0.15^ya^	3.5 ± 0.23^xy^	3.7 ± 0.16^yz^
Met-enkephalin	3.4 ± 0.16^x^	3.5 ± 0.16^x^	3.9 ± 0.11^ya^	3.8 ± 0.15^y^	3.8 ± 0.11^y^
Stress (+ vehicle)	3.6 ± 0.13^x^	3.9 ± 0.16^y^	4.5 ± 0.11^zb^	4.5 ± 0.24^z^	4.0 ± 0.13^y^
Stress + Met-enkephalin	3.6 ± 0.16^x^	3.8 ± 0.08^y^	4.1 ± 0.18^zb^	3.9 ± 0.19^y^	3.6 ± 0.14^x^
**Reverse T_3_ (rT_3_) nmoles L^−1^**					
Control (vehicle)	42.6 ± 0.68	43.8 ± 0.66	42.4 ± 0.51	41.8 ± 0.86	43.6 ± 0.51
Met-enkephalin	44.2 ± 1.15^y^	45.4 ± 0.68^z^	43.0 ± 0.71^xy^	40.0 ± 1.05^x^	41.8 ± 0.73^x^
Stress (+ vehicle)	45.6 ± 0.75^y^	50.8 ± 1.06^z^	41.2 ± 0.86^x^	38.0 ± 0.71^w^	44.4 ± 0.87^y^
Stress + Met-enkephalin	44.5 ± 0.50^y^	44.8 ± 1.16^y^	42.8 ± 0.66^y^	40.6 ± 0.93^x^	40.0 ± 1.38^x^

^v, w, x, y, z^Different letters indicate difference (*P* < 0.05) across rows (with time).

^a, b, c, d^Different letters indicate difference (*P* < 0.05) between treatments.

**Fig. 1:**
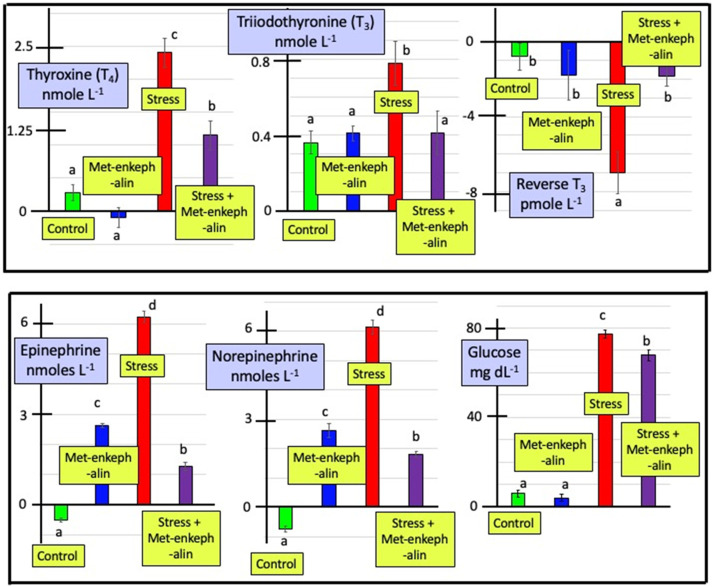
Effect of Met-enkephalin and/or restraint stress on the increase or decrease (Δ) in plasma concentrations of thyroid hormones, catecholamines and glucose (expressed as the plasma concentration 15 min after injection or subjection to stress minus the mean of the pre-treatment concentrations). Top: increase or decrease (Δ) in plasma concentrations of epinephrine, norepinephrine and glucose. Bottom: increase or decrease (Δ) in plasma concentrations of thyroxine (T_4_), triiodothyronine (T_3_) and reverse T_3_ (rT_3_).

The data were also analyzed as delta (increase/decrease) in plasma concentration of catecholamines (*i.e.* increase/decrease) (see Fig. 1). Delta plasma concentrations of either EP or NE were increased (*P* < 0.001) following Met-enkephalin administration (Fig. 1). Greater increases (*P* < 0.001) were observed in pullets subjected to restraint stress (Fig. 1). Met-enkephalin administration greatly attenuated (*P* < 0.001) the effects of restraint stress on delta plasma concentrations of both EP or NE (Fig. 1). There were increases, albeit transitory, in plasma concentrations of EP and NE in chickens subjected to restraint stress (Fig. 1). This is consistent with the “fight or flight syndrome” (reviewed: Goldstein, 2010; Dennis, 2016). The increase in plasma concentrations of EP and NE in restrained stressed chickens is new but to be expected. A novel finding is that peripherally administered Met-enkephalin markedly attenuated the increase in plasma concentrations of EP and NE in stressed chickens. In contrast, plasma concentrations of EP and NE were elevated following peripheral administration of Met-enkephalin (Fig. 1). This is consistent with Met-enkephalin being a component in the stress response but also modulates the response, possibly to prevent over-reaction.

### Met-enkephalin and/or restraint stress on plasma concentrations of glucose

In 14-week-old pullets subjected to restraint stress for 15 min, plasma concentrations of glucose were increased by 39.9 % (*P* < 0.001) compared to control birds (Table 1). Met-enkephalin administration had no effect on plasma concentrations of glucose (Table 1).

### Met-enkephalin and/or restraint stress on plasma concentrations of thyroid hormones

There were increases (*P* < 0.001) in the plasma concentrations of T_4_ (by 9.6 %) and T_3_ (by 25 %, in pullets subjected to restraint stress but little effect on plasma concentrations of rT_3_ (Table 1). When data are expressed as delta plasma concentrations of T_4_ or T_3_, there were clearly increases (*P* < 0.001) in stressed pullets (Fig. 1). Moreover, stress was accompanied by decreases (*P* < 0.001) in plasma concentrations of rT_3_ (Fig. 1). While Met-enkephalin administration *per se* was without effect on delta plasma concentrations of T_4_ or T_3_ or rT_3_, Met-enkephalin decreased (*P* < 0.001) the effects of restraint stress (Fig. 1).

There were increases in the plasma concentrations of both T_3_ and T_4_ in chickens subjected to restraint stress (Fig. 1, Table 1). The former is similar to the effects of other stressors including fasting (Van der Geyten et al., 19990 and heat stress (Beckford et al., 2020). Unexpectedly, the stress induced increases in plasma concentrations of both T_3_ and T_4_ were markedly attenuated in birds receiving Met-enkephalin administration peripherally (Fig. 1, Table 1). This represents a novel observation for any species. Moreover, this suggests that Met-enkephalin may play a role in stress preventing over-shooting and facilitating an optimal response. Plasma concentrations of T_3_, T_4_, and rT_3_ were unaffected by the peripheral administration of Met-enkephalin alone (Fig. 1, Table 1).

### Effects of repeated blood sampling

Effects of repeated blood sampling were examined by comparing the sampling at 5 and 1 min prior to imposition of treatments. Unexpectedly, there were increases, albeit small, in the plasma concentrations of EP (by 7.2 %, *P* < 0.001), NE (by 3.6 %, *P* < 0.01), glucose (by 2.0 %, *P* < 0.05) T_4_ (by 4.2 % *P* < 0.001), T_3_ (by 5.5 %, *P* < 0.01) and rT_3_ (by 4.5 %, *P* < 0.05).

### Relationships

There was a very strong relationship between plasma concentrations of EP and NE [adjusted R^2^ 0.951 (*P* = 2.08E^−13^). This does not appear to be consistent with the supposed different cellular origins of EP (chromaffin cells) and NE (sympathetic nervous system) (Dennis, 2016). There were strong relationships [adjusted R^2^ >0.500 (*P* < 0.0002)] between plasma concentrations of T_3_ and those of T_4_ or glucose or EP or NE and between plasma concentrations of T_4_ and those of glucose

In conclusion, the opioid peptide, Met-enkephalin, influenced adrenal medulla activity increasing the plasma concentrations of catecholamines *per se* but did not change those of thyroid hormones. However, the stress responses of increased plasma concentrations of both catecholamines and thyroid hormones were significantly attenuated by a single injection of Met-enkephalin. Plasma level of glucose was also slightly decreased by a concomitant application of restraint and opioid peptide.

## Funding

This work was financially supported by the subvention of the Ministry of Science and Higher Education to the University of Agriculture in Krakow, Poland (subvention number 020002/D015/2025 year).

## CRediT authorship contribution statement

**Colin G. Scanes:** Writing – review & editing, Writing – original draft, Validation, Methodology, Investigation, Formal analysis, Conceptualization. **Krystyna Pierzchala-Koziec:** Writing – review & editing, Writing – original draft, Validation, Supervision, Resources, Project administration, Methodology, Investigation, Funding acquisition, Formal analysis, Data curation, Conceptualization.

## Disclosures

The authors declare that there are no conflicts of interest.
